# Botulinum Toxin for Temporomandibular Disorder: Current Evidence, Clinical Applications, and Future Perspectives

**DOI:** 10.3390/toxins18100419

**Published:** 2026-09-29

**Authors:** Martina Ferrillo, Claudio Curci, Dario Calafiore, Lorenzo Lippi, Yasemin Yumuşakhuylu, Michele Vecchio, Vincenzo Quinzi, Alfredo De Rosa, Marco Invernizzi, Alessandro de Sire

**Affiliations:** 1 Dentistry Unit, Department of Health Sciences, University of Catanzaro “Magna Graecia”, 88100 Catanzaro, Italy; martina.ferrillo@unicz.it; 2 Physical Medicine and Rehabilitation Unit, Department of Neurosciences, ASST Carlo Poma, 46100 Mantova, Italy; dario.calafiore@asst-mantova.it; 3 Physical and Rehabilitative Medicine, Department of Health Sciences, University of Eastern Piedmont “A. Avogadro”, 28100 Novara, Italy; lorenzo.lippi@uniupo.it (L.L.); marco.invernizzi@med.uniupo.it (M.I.); 4 Dipartimento Attività Integrate Ricerca e Innovazione (DAIRI), Translational Medicine, Azienda Ospedaliero-Universitaria (AOU) SS. Antonio e Biagio e Cesare Arrigo, 15121 Alessandria, Italy; 5 Faculty of Medicine, Department of Physical Medicine and Rehabilitation, Istanbul Medeniyet University, 34700 Istanbul, Turkey; yasemin.yumusakhuylu@medeniyet.edu.tr; 6 Department of Biomedical and Biotechnological Sciences, University of Catania, 95124 Catania, Italy; michele.vecchio@unict.it; 7 Physical Medicine and Rehabilitation Unit, AOU Policlinico G. Rodolico, University of Catania, 95124 Catania, Italy; 8 Department of Life Science, Health and Health Professions, Link Campus University, 00165 Rome, Italy; v.quinzi@unilink.it (V.Q.); a.derosa@unilink.it (A.D.R.); a.desire@unilink.it (A.d.S.)

**Keywords:** botulinum neurotoxin type A, temporomandibular disorders, myofascial pain, masticatory muscles, orofacial pain, rehabilitation, bruxism, adverse effects

## Abstract

Temporomandibular disorders (TMDs) are heterogeneous musculoskeletal conditions frequently characterized by pain, impaired mandibular function, and reduced quality of life. Botulinum neurotoxin type A (BoNT-A) has emerged as a potential therapeutic option, particularly for persistent myogenous TMDs associated with masticatory muscle hyperactivity. This narrative review critically examines the mechanisms of action, current clinical evidence, therapeutic applications, and safety profile of BoNT-A in TMD management. Available evidence suggests that BoNT-A can reliably reduce masticatory muscle activity and may provide pain relief in selected patients with refractory myogenous TMDs; however, its superiority over placebo or established conservative treatments remains inconsistent. Considerable heterogeneity in patient selection, injected muscles, dosing regimens, injection techniques, and outcome assessment currently prevents the definition of standardized treatment protocols. Although short-term adverse events are generally mild and transient, repeated injections may induce muscle atrophy, reduced bite force, and potentially mandibular skeletal changes, whose long-term clinical relevance remains uncertain. Therefore, BoNT-A should not be considered a first-line treatment but rather a carefully selected adjunct within a multimodal rehabilitation strategy after appropriate conservative management has failed. Future phenotype-stratified comparative trials with standardized protocols and long-term structural safety outcomes are warranted.

## 1. Introduction

Temporomandibular disorders (TMD) include a heterogeneous group of musculoskeletal and neuromuscular conditions affecting the temporomandibular joint (TMJ), the masticatory muscles, and the associated periarticular structures, generally classified into joint-related (arthrogenous) and muscle-related (myogenous) subtypes according to the Diagnostic Criteria for Temporomandibular Disorders (DC/TMD) [1,2]. In this context, Myogenous TMDs, originated primarily by muscle hyperactivity and myofascial pain, are considered particularly prevalent with a very high disability status, frequently coexisting with tension-type headache, bruxism, cervical pain, and reduced oral-health-related quality of life [3,4]. The clinical burden extends beyond localized pain, affecting mastication, speech, sleep quality, and psychosocial wellbeing. Moreover, TMD-related chronic pain is increasingly recognized as a major cause of healthcare utilization and productivity loss [5,6]. Given the multifactorial biopsychosocial etiology of TMDs, management requires an individualized approach integrating rehabilitative, behavioral, pharmacological, and, in selected cases, minimally invasive interventions [7].

For the purposes of this narrative review, the literature was identified through searches of PubMed and Scopus covering published papers up to 2026 using combinations of terms related to “temporomandibular disorders,” “myogenous TMD,” “myofascial pain,” “bruxism,” “botulinum toxin,” “botulinum neurotoxin type A,” “masticatory muscles,” “masseter,” “temporalis,” “pain,” “mandibular function,” and “safety.” Reference lists of relevant reviews and clinical studies were also screened to identify additional publications. Particular attention was paid to randomized controlled trials, systematic reviews, meta-analyses, and recent clinical and anatomical studies published between 2024 and 2026. Because this was a narrative rather than a systematic review, references were selected according to their clinical relevance, methodological contribution, and ability to address the principal questions concerning efficacy, treatment protocols, patient selection, and safety; no formal risk-of-bias assessment or meta-analytic study selection procedure was performed.

Multimodal conservative management of TMD patients plays a central role in TMD treatment, requiring specific knowledge to combine different therapeutic approaches such as occlusal splint therapy, physical therapy, pharmacotherapy, and behavioral therapy [8,9]. Manual therapy and therapeutic exercise, including jaw mobilization and postural rehabilitation, have shown moderate short-term benefit on pain and mandibular function, although the quality of supporting evidence remains heterogeneous and long-term efficacy is not firmly established [10]. Pharmacological approaches, such as nonsteroidal anti-inflammatory drugs, muscle relaxants, and low-dose tricyclic antidepressants, may provide symptomatic relief but are limited by modest effect sizes, side effects, and the risk associated with prolonged use, particularly in chronic presentations [11]. Nowadays, behavioral interventions such as cognitive-behavioral therapy, biofeedback, and relaxation techniques address the psychosocial component of TMD but require sustained patient engagement and specialized resources, with evidence of a persistent advantage therapy still limited [12]. A relevant subset of patients, specifically those with refractory myofascial pain or bruxism-related muscle hyperactivity, experience insufficient or transient benefit from these conservative approaches and remain symptomatic despite multimodal management [13,14]. These constraints are particularly evident in patients with high-frequency parafunctional activity, such as severe bruxism, in whom mechanical off-loading strategies alone are often insufficient to interrupt the underlying pattern of muscle overactivity [15]. In summary, this unmet clinical need has stimulated growing interest in adjunctive, minimally invasive therapies capable of directly targeting muscle hyperactivity, among which intramuscular botulinum neurotoxin type A (BoNT-A) injections have emerged as a promising option [16,17].

Botulinum toxin-type A (BoNT-A) is a neurotoxin produced by Clostridium botulinum that inhibits acetylcholine release at neuromuscular junctions by binding to presynaptic cholinergic and nerve terminals, which results in muscle relaxation and reduced pain [18,19,20]. By inhibiting acetylcholine exocytosis, BoNT-A produces a dose-dependent, reversible chemodenervation that interrupts the sustained muscle fiber contraction, thereby restoring local blood flow, reducing metabolic demand, and theoretically breaking the vicious cycle of contraction–ischemia–pain [17].

The resulting reduction in muscle activity is a well-established pharmacological effect, whereas its translation into clinically meaningful improvements in pain, mandibular function, or quality of life is less consistent across TMD studies.

Beyond its peripheral neuromuscular effect, several evidence suggests that BoNT-A also exerts direct antinociceptive and anti-inflammatory actions independent of muscle relaxation [21]. From a clinical perspective, BoNT-A injection into trigger points offers several theoretical advantages over other interventional approaches [22].

## 2. Mechanism of Action of BoNT-A in TMD

### 2.1. Neuromuscular Blockade and Reduction in Muscle Hyperactivity

Furthermore, careful attention to injection depth and localization is required to avoid unwanted diffusion toward adjacent muscles involved in mastication, swallowing, or facial expression, since excessive weakening of these structures may result in functional impairment rather than therapeutic benefit, an aspect that continues to drive interest in ultrasound-guided injection techniques [23].

Importantly, reduction in electromyographic activity or bite force should be regarded as evidence of pharmacological neuromuscular inhibition rather than as a surrogate for clinical efficacy. A reduction in muscle activity may coexist with improvement in pain in some patients, but the relationship between these outcomes is neither linear nor consistently demonstrated.

### 2.2. Potential Antinociceptive Effect

**Figure 1:**
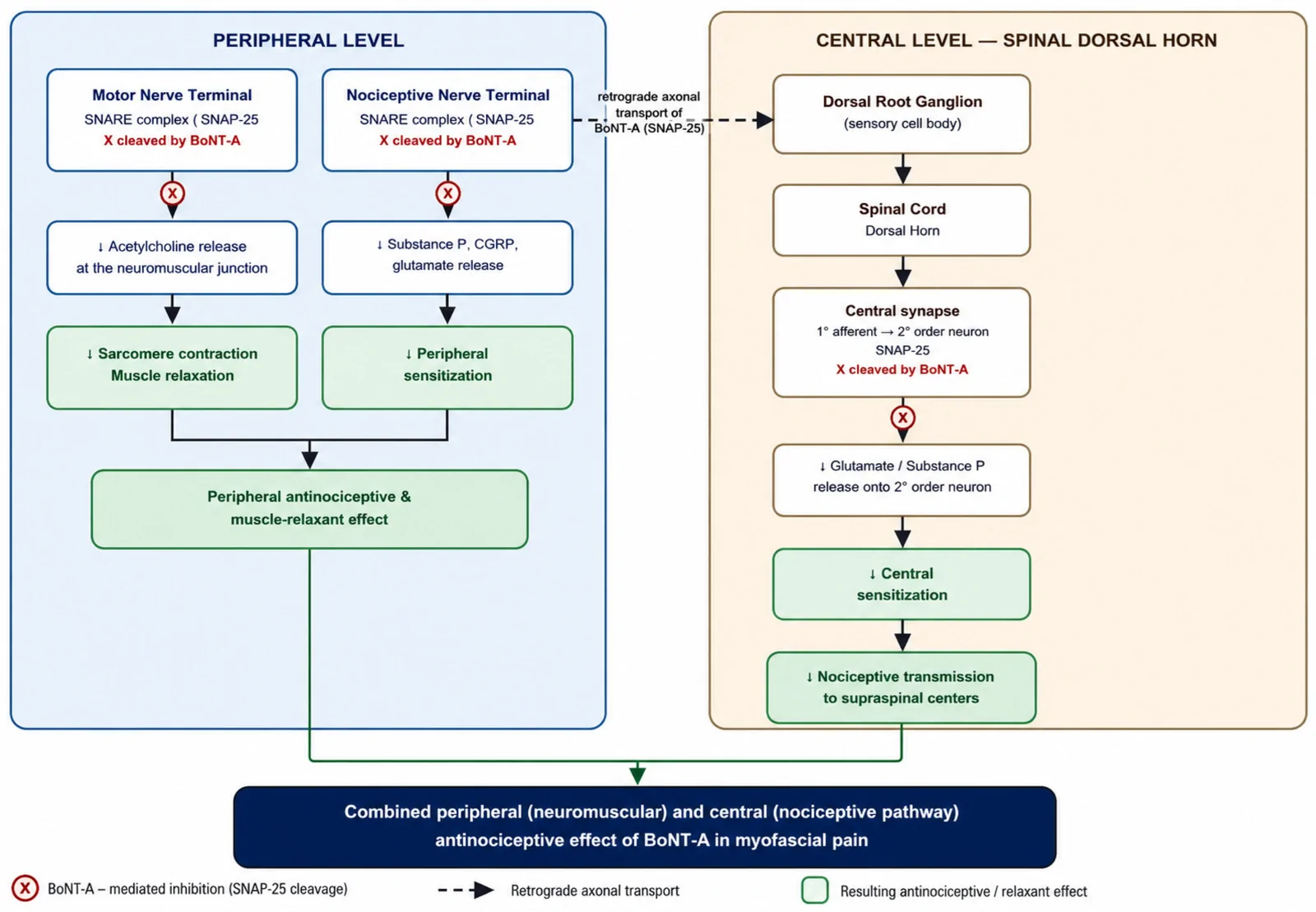
Potential antinociceptive effect of BoNT-A.

In addition to direct action at the neuromuscular junction, preclinical and clinical evidence indicates that BoNT-A exerts an independent antinociceptive and anti-inflammatory effect that cannot be fully explained by muscle relaxation alone [24]. At the peripheral level, BoNT-A has been shown to inhibit the SNARE-dependent release of pro-algesic and pro-inflammatory mediators from sensory nerve terminals, including glutamate, substance P, and calcitonin gene-related peptide (CGRP), thereby reducing peripheral sensitization of nociceptors within the injected muscle (see Figure 1) [25,26].

These mechanistic findings provide biological plausibility for an analgesic effect, but they should not be interpreted as proof of clinical superiority in patients with TMD.

Moreover, CGRP release occurs in the meninges and the trigeminal caudal nucleus and is amplified by P2Y purinergic receptors on satellite glial cells, which are influenced by factors such as 17-beta-estradiol [27]. Conversely, P2X3 receptor-mediated peripheral pain signaling in neuronal afferents can attenuate CGRP activity [28].

### 2.3. Rationale for Use in Myogenous TMDs and Bruxism-Associated Symptoms

Myogenous temporomandibular disorders (M-TMDs) constitute one of the most common TMD subtypes and are clinically defined by masticatory myofascial pain, restricted mandibular excursion, and muscle tenderness on palpation [20]. This parafunctional hyperactivity is widely regarded as a key risk factor for the onset and perpetuation of M-TMD symptoms, establishing a direct pathophysiological continuum between the two conditions and providing the biological basis for a shared pharmacological target [29].

However, bruxism, myofascial TMD, and masseter hypertrophy should not be considered interchangeable clinical entities: the therapeutic objective in bruxism is primarily reduction in excessive muscle activity, whereas treatment of myogenous TMD aims principally to reduce pain and disability while preserving adequate mandibular function.

The clinical evidence supporting this rationale has grown substantially in recent years. Careful patient selection, informed by a thorough clinical and instrumental characterization of the muscular phenotype, therefore represents a key determinant of treatment success and should guide future protocol standardization efforts [30]. Lastly, despite an expanding body of clinical evidence, the use of BoNT-A in TMD remains characterized by heterogeneous injection protocols, variable dosing regimens, and an incomplete understanding of its precise mechanisms of action at the neuromuscular junction and along nociceptive pathways [16,31,32].

## 3. Clinical Evidence: BoNT-A for Myogenous TMD

### 3.1. Main Randomized Controlled Trials, Systematic Reviews and Meta-Analyses

The first clinical studies of BoNT-A in TMD were largely uncontrolled or involved small samples. In particular, Freund et al. reported improvement in pain, function, mouth opening and tenderness following injection of temporalis muscles with 25 units each, although the study lacked a control group [33].

Subsequent controlled studies provided more informative but still inconsistent findings. Guarda-Nardini et al. conducted a double-blind placebo-controlled pilot study in 20 patients with bruxism and myofascial pain and reported greater improvement in pain and mandibular function in the BoNT-A group [34].

By contrast, the randomized controlled trial by Nixdorf et al. in patients with chronic myogenous orofacial pain did not establish convincing superiority of BoNT-A over placebo [35]. Similarly, Ernberg et al. found no clear clinically relevant benefit in persistent myofascial TMD pain in a multicenter randomized trial [36]. These studies also differed substantially in patient phenotype, treated muscles, toxin formulation, dosing, comparator, and outcome assessment, making direct comparison difficult.

These apparently contradictory findings illustrate the central problem of the BoNT-A literature: the intervention is relatively standardized pharmacologically (although not regarding muscles and dosages), but the disease phenotype and patient characteristics are heterogeneous and might influence the outcomes.

Later trials have produced a more favorable signal in selected populations. Kurtoglu et al. studied patients with myofascial pain with or without disc displacement and reported improvements following BoNT-A treatment with injection of temporalis and masseter muscles (20 and 30 UI) [37]. Guarda-Nardini et al. subsequently compared BoNT-A with fascial manipulation in patients with myogenous TMD and reported short-term improvement in myofascial pain following both interventions [38].

A randomized trial involving 80 women with persistent myofascial pain compared three BoNT-A doses with saline and found improvements in mandibular range of motion and muscle tenderness at 180 days of follow-up [39]. However, the study was not designed to establish a dose–response relationship, and all active-treatment groups received injections into the masseter and anterior temporalis muscles. Accordingly, the doses evaluated in this study should be interpreted as protocol-specific study doses rather than as evidence of an optimal therapeutic dose.

A further randomized study of 60 patients with localized or referred masticatory myofascial pain compared BoNT-A with saline and lidocaine. BoNT-A produced greater improvements in pain and mandibular movement over the follow-up period [40].

More recently, Kim et al. conducted a double-blind placebo-controlled pilot trial involving 21 patients with myogenous TMD and headache. BoNT-A was injected into tender areas involving masseter, temporalis, splenius capitis, sternocleidomastoid, and trapezius muscle.

However, because several anatomical targets were treated simultaneously, the study cannot determine whether the observed clinical response was attributable to treatment of the masticatory muscles, the cervical muscles, or their combined effect.

These findings suggest that BoNT-A may be particularly useful when TMD is part of a broader muscular pain phenotype involving headache and multiple tender muscles.

The evidence becomes more informative when randomized trials are considered collectively.

A systematic review and meta-analysis published in 2020 included 12 randomized controlled trials and found that BoNT-A was slightly more effective than placebo for pain reduction at one month, but significant differences were not maintained consistently at three or six months. The analysis also reported variable comparisons with conventional treatments and physical interventions [41].

A subsequent review of randomized trials identified 24 RCTs published through 2021. Nine investigated myofascial pain, four articular TMD, eight bruxism and three masseter hypertrophies. The review concluded that evidence was relatively favorable for masseter hypertrophy but remained equivocal for myogenous TMD and limited for articular disorders [42]. The 2024 meta-analysis by Saini et al. included 14 RCTs and 395 patients and found evidence suggesting improvement in several TMD-related outcomes after BoNT-A, although substantial heterogeneity was present, with no statistical differences in pain and mouth opening [43]. Li et al. subsequently analyzed 15 randomized trials involving 504 participants with muscular TMD. Their findings supported reductions in pain and improvements in selected functional outcomes, but again emphasized heterogeneity in treatment protocols, diagnostic criteria and outcome measures [44], suggesting that a bilateral dose of 60–100 U of BoNT-A might be an optimal choice for treating muscular TMD pain. However the proposed bilateral dose range of 60–100 U should be considered a range observed within the included literature rather than an established therapeutic recommendation. Furthermore, dose comparisons should not be extrapolated across different BoNT-A formulations because units are formulation-specific and are not directly interchangeable.

The 2024 umbrella review by De la Torre Canales et al. [45] provides perhaps the most useful synthesis of the pre-2024 evidence; no convincing superiority was found for mandibular movement, and concerns were raised regarding adverse effects involving muscle and bone [45].

The newest evidence is particularly relevant. The 2026 systematic review by de Santana et al. [46] specifically examined BoNT-A for myogenous TMD, focusing on duration and dosage parameters. Ten randomized clinical trials were included. Its findings further emphasize that the available evidence remains heterogeneous with respect to dose, injection protocol, follow-up, and clinical outcomes, and therefore does not establish a universally applicable dosing regimen.

### 3.2. Effects on Pain, Mandibular Function and Quality of Life

The clinical relevance of BoNT-A in TMD should be considered across three distinct but interrelated domains: pain, mandibular function and quality of life.

Pain remains the outcome for which the clinical rationale for BoNT-A is strongest, particularly in patients with persistent myogenous TMD and a clearly identifiable muscular pain component.

The relationship between BoNT-A and mandibular function is considerably more complex [47].

Although BoNT-A consistently reduces masticatory muscle activity and bite force, these effects are not inherently beneficial and may become clinically undesirable if excessive. The therapeutic objective should therefore not be maximal suppression of muscle activity, but rather a clinically useful reduction in pathological muscular loading while preserving adequate mastication, mandibular mobility, and facial function.

**Figure 2:**
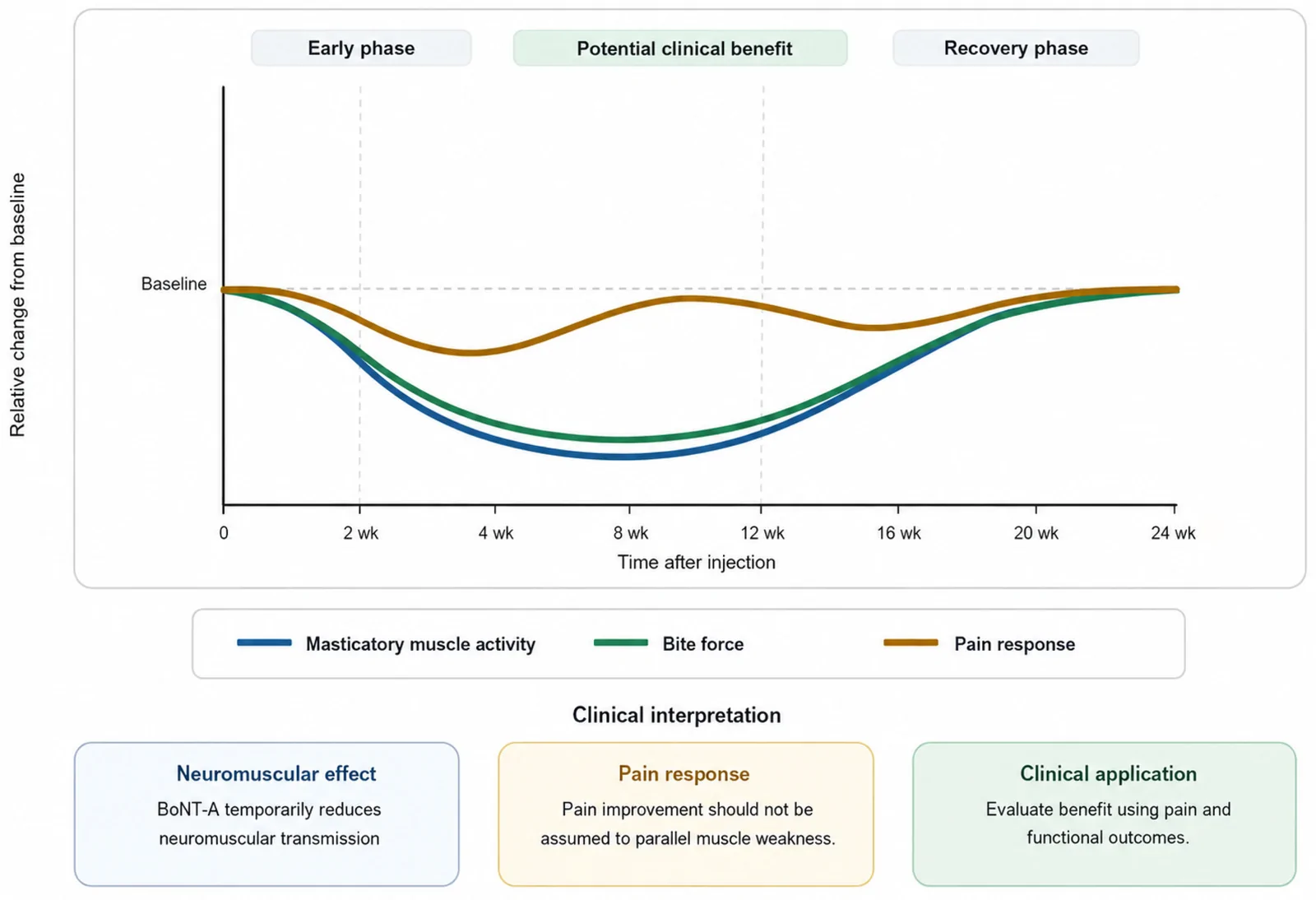
Conceptual temporal relationship between BoNT-A effects and clinical response.

Improvements in quality of life have been reported following BoNT-A, particularly in patients with myogenous TMD who experience substantial baseline pain and functional limitation. Prospective studies using disease-specific or generic patient-reported outcome measures have documented improvements in oral health-related quality of life after treatment [48,49,50]. However, these patient-reported improvements should be interpreted as clinical outcomes in their own right and should not be inferred solely from reductions in muscle activity or bite force. See Figure 2 for further details.

### 3.3. Main Limitations and Heterogeneity of Current Evidence

Several methodological limitations should be considered when interpreting the clinical evidence for BoNT-A in TMD. First, substantial heterogeneity exists in the selection and measurement of clinical outcomes. Across TMD intervention trials, pain, mandibular function, psychosocial outcomes and adverse events have been assessed using a wide range of instruments, while some clinically relevant domains have been inconsistently reported, limiting direct comparison between studies [51]. Second, the intervention itself remains insufficiently standardized.

Differences in toxin formulation, target muscles, total dose, dose per muscle, number and location of injection points, anatomical guidance, comparator interventions, and follow-up duration substantially limit the comparability of published results.

A further limitation is the frequent conflation of biological and clinical outcomes. Reductions in electromyographic activity, bite force, or muscle thickness demonstrate a pharmacological or structural effect of BoNT-A but do not necessarily establish improvement in pain, mandibular function, or quality of life. Future studies should therefore report these outcomes separately and prospectively define clinically meaningful thresholds for patient-reported benefit.

## 4. Clinical Applications and Treatment Considerations

### 4.1. Patient Selection, Particularly Refractory Myogenous TMDs

Randomized and prospective studies conducted in patients with persistent myofascial TMD have generally selected relatively homogeneous populations characterized by muscular pain and tenderness, providing the strongest biological rationale for BoNT-A in this subgroup [52,53].

The clinical phenotype should therefore be characterized beyond the presence of muscular tenderness.

Recent evidence specifically examining myogenous TMD suggests that greater pain reductions may be observed in refractory myofascial populations, although the small number of studies and methodological heterogeneity prevent this observation from being considered a validated responder characteristic [46].

Accordingly, the most defensible candidate for BoNT-A is a patient with persistent predominantly myogenous TMD, reproducible familiar muscular pain, a demonstrable relationship between symptoms and muscle loading, clinically meaningful pain or functional impairment, and an inadequate response to appropriately delivered conservative treatment [17]. This phenotype should currently be regarded as a pragmatic clinical selection model rather than a validated predictor of response.

### 4.2. Target Muscles, Doses and Injection Techniques

Across clinical trials, substantial variability has been reported in the muscles injected, total dose, dose distribution, number of injection points and anatomical approach, limiting direct comparison between studies and preventing the establishment of a universally accepted treatment protocol [43,44,45]. Reported doses should therefore be understood as protocol-specific ranges observed in the literature rather than established therapeutic recommendations.

The masseter is the most consistently investigated target and is included in virtually all contemporary randomized trials of myogenous TMD, reflecting its major contribution to mandibular force generation and its accessibility for intramuscular injection [39,54,55]. The anterior temporalis is frequently treated concomitantly, particularly when clinical examination demonstrates reproducible familiar pain or tenderness in this muscle [56].

A 2024 systematic review and meta-analysis found that most randomized trials administered BoNT-A to the masseter, while the temporalis was included in a substantial proportion of studies; medial and lateral pterygoid injections were considerably less frequent [43].

Similarly, a survey of clinicians treating TMD-associated myalgia demonstrated considerable variation in both target muscles and dosing practices, with the masseter being the most commonly injected muscle and reported onabotulinumtoxinA doses ranging from 30 to 100 U per masseter [57]. This range reflects reported clinical practice and should not be interpreted as an evidence-based recommended dose. Importantly, units reported for onabotulinumtoxinA, abobotulinumtoxinA, and incobotulinumtoxinA should not be directly converted or considered equivalent across formulations.

This marked variability reflects the absence of an evidence-based consensus rather than established differences in therapeutic requirements.

The pilot trial by Kim et al. illustrates the methodological problem associated with injecting multiple muscles simultaneously: although clinical improvement occurred, it is impossible to determine which anatomical target was responsible for the effect [54].

Contemporary protocols have generally used multiple small-volume injections within the masseter and temporalis rather than concentrating the entire dose at a single site [58]. In this recent prospective pilot study, 50 U of onabotulinumtoxinA was distributed bilaterally between the masseter and anterior temporalis, using five masseter injection points and two anterior temporalis points per side; sites were identified through palpation and contraction during dental occlusion.

The authors deliberately excluded the lateral pterygoid because of its deep anatomical position and proximity to major vascular and orbital structures, emphasizing the greater reproducibility of masseter and temporalis injections in routine clinical practice; although the lateral pterygoid may be relevant in selected muscular phenotypes, its anatomical depth and proximity to critical structures make its injection technically more demanding and less readily standardized [59,60].

Anatomical guidelines published in 2022 proposed more precise injection zones for the masseter and temporalis based on cadaveric dissection and motor endplate distribution [61]. More recent sonoanatomical work has further highlighted the potential value of ultrasonography for identifying muscle layers, anatomical boundaries and adjacent structures, particularly in anatomically complex facial regions [62]. A 2026 cadaveric study of the masseter identified a relatively safer triangular region with lower probability of encountering the facial nerve, parotid gland, parotid duct and facial vessels, and proposed a three-point injection approach [63].

Ultrasound guidance may be particularly relevant when deeper muscles are targeted or when anatomical variability is substantial.

Experimental and clinical evidence suggests that BoNT-A can reduce muscle volume, alter muscle activity and potentially influence mandibular bone remodeling [64,65,66,67]. These findings should be distinguished according to the level of evidence: experimental models establish biological plausibility, observational and clinical studies document structural changes in treated patients, whereas evidence that such changes translate into clinically meaningful skeletal harm remains limited and inconsistent.

Although the clinical significance of these skeletal changes remains uncertain, these findings provide an additional argument against indiscriminate treatment of multiple muscles or repeated escalation of dose in patients with an inadequate response.

### 4.3. Role of BoNT-A Within a Multimodal Rehabilitation Approach

Contemporary rehabilitation approaches instead combine therapeutic exercise, manual therapy, education, self-management and, when appropriate, behavioural interventions [10,68,69,70,71]. Exercise-based interventions have demonstrated improvements in pain and active and passive mouth opening, while manual therapy may provide additional benefits for pain, mandibular mobility and disability [72].

Evidence from multimodal physiotherapy studies indicates that programmes combining exercise with manual or physical modalities may produce greater benefits than exercise alone in selected myogenous TMD populations [73,74,75,76].

Accordingly, BoNT-A should be considered an adjunct to, rather than a replacement for, an individualized rehabilitation programme. Therapeutic exercise, education regarding jaw use and parafunctional behaviors, self-management strategies, manual therapy when indicated, and behavioural interventions should remain integrated according to the patient’s clinical and psychosocial profile.

In this framework, BoNT-A may be considered when persistent muscular pain or clinically significant muscle hyperactivity remains despite appropriately delivered conservative management. Following injection, rehabilitation should continue with the aim of maintaining mandibular mobility, restoring functional muscle coordination, addressing parafunctional behaviors, and consolidating self-management strategies. The available evidence does not support withdrawing these interventions and using BoNT-A as stand-alone therapy.

The evidence grading presented in Table 1 represents an interpretive synthesis by the present authors based on the consistency, magnitude, and limitations of the available literature. It is not a formal GRADE assessment or a validated systematic-review evidence-certainty evaluation. The categories are therefore intended to facilitate clinical interpretation rather than to represent formal levels of certainty. See Table 1 for further details.

## 5. Safety Profile and Adverse Events

Botulinum neurotoxin type A (BoNT-A) is generally described as well tolerated when injected into the masticatory muscles, but this conclusion rests on small trials, single treatment cycles and short follow-up, and is further weakened by heterogeneity in formulation, dose, target muscles, injection technique and adverse-event ascertainment [77]. Meta-analyses have not demonstrated a clear excess of short-term adverse events over placebo, although the certainty of that finding is low and reporting has been largely passive [43,78]. The underlying difficulty is pharmacological. Two registers should therefore be kept apart: transient, largely technique-dependent effects that resolve within weeks, and dose- and repetition-dependent structural effects on muscle and bone that may not [79].

Across randomized controlled trials, adverse events attributed to BoNT-A injection into the masticatory muscles are consistently described as mild, self-limiting and of short duration: injection-site pain and local tenderness most frequently, followed by transient headache, a subjective sensation of weakness during chewing, dysphagia and cosmetic alteration of the smile [77,78,80]. Rarer events reflect unintended diffusion rather than the drug itself: transient paralysis of the risorius muscle has been described after standard bilateral masseter and anterior temporalis injection in a patient enrolled in a clinical trial [81]. Although generally transient, these events should not be considered clinically trivial because impairment of chewing, swallowing, speech, or facial expression can meaningfully affect patients.

These descriptions capture only part of the safety question. Reviews examining objective functional outcomes rather than patient-reported events describe a more consistent signal: electromyographic activity was reduced in every study that measured it, and occlusal force in all four studies that assessed it [82]. In a randomized, placebo-controlled cross-over trial using a moderate dose of 50 U, BoNT-A produced a significant decline in muscle performance while its analgesic effect relative to saline remained equivocal [83]. This distinction is important because a measurable reduction in muscle performance represents a pharmacological effect that may be desirable in selected hyperactivity phenotypes but may also constitute an adverse functional effect when excessive.

Muscle atrophy follows a reproducible time course. Magnitude depends on indication and dose: in aesthetically motivated masseter treatment, where higher doses are customary, mean thickness falls by approximately 31% at three months [84]. In a randomized triple-blinded trial, a single session of abobotulinumtoxinA produced changes that had largely resolved by six months, whereas a second session three months later left masseter thickness and electromyographic activity significantly reduced at six months, leading the authors to conclude that multiple injections cause functional adverse effects [27].

These findings demonstrate that recovery after a single exposure cannot be extrapolated to repeated treatment cycles, particularly when intervals between injections are short or cumulative exposure is increased.

An underappreciated consequence is compensatory redistribution of masticatory load. Prospective ultrasonography after masseter injection has shown a significant increase in temporalis thickness at rest and in contraction, inversely related to the decrease in the masseter [85], and a randomized triple-blinded trial documented secondary changes in the non-injected temporalis [86].

Reversibility offers partial reassurance, but only for the exposure actually studied. In a controlled clinical trial with an exceptionally long observation window, masseter thickness was significantly reduced at one and three months after a single application yet did not differ from baseline at 72 months, while the analgesic benefit was maintained [87].

However, recovery following a single treatment cycle does not establish that structural or functional changes induced by repeated cycles are equally reversible, and long-term data on cumulative exposure in patients with TMD remain limited.

Atrophy and analgesia are in any case dissociated: pain intensity decreases before muscle thinning is fully established, and correlations between thickness and pain are absent at most time points and anatomical sites [88]. That is the direct pharmacological argument for the lowest effective dose.

The potential effect of masticatory-muscle chemodenervation on mandibular bone is the most consequential unresolved safety issue in this field, and the concern rests on a well-established principle: BoNT-induced paralysis is a standard experimental model of disuse-related bone loss, and the masticatory muscles are the principal source of mechanical loading of the mandible [89]. These experimental observations establish biological plausibility but do not, by themselves, demonstrate clinically relevant bone loss in patients receiving BoNT-A for TMD.

Human evidence is more equivocal, and its most alarming signal comes from the smallest and least controlled studies. A randomized trial comparing three doses in persistent myofascial pain likewise identified dose-related decreases in coronoid and condylar process bone volume among its prespecified adverse effects [90].

Larger and better-controlled studies have not reproduced these findings. In a phantom-calibrated comparison of 35 women who had received at least two treatment cycles with 44 who had not, mean bone density and condylar volume did not differ between groups; the only signal was an inverse relationship between temporalis dose and trabecular density of the mandibular body within the exposed cohort [91].

Notably, the more pronounced safety signals have generally arisen from the smaller and less controlled studies, whereas the two largest and better-controlled human datasets have been more reassuring [91,92]. This is not the same as evidence of long-term safety, for three reasons. Second, it was conducted in healthy adults with masseter muscle prominence rather than in patients with TMD, was limited to two treatment cycles and followed participants for 12 months [92].

Almost every uncertainty described above is amplified by the absence of an agreed treatment protocol. Unsurprisingly, the most recent systematic review of randomized trials concluded that the evidence is insufficient to determine whether a clinically relevant dose–response relationship exists across the regimens evaluated [77].

The consequences are more serious for safety than for efficacy. Standardization is therefore not a reporting preference but a precondition for knowing whether a reproducible therapeutic window exists at all.

That standardization is achievable has nevertheless been demonstrated. In a retrospective series of 98 adults with DC/TMD-confirmed myogenous TMD refractory to at least three months of conservative management, a predefined incobotulinumtoxinA protocol was associated with a favourable outcome in 79.6% of patients at 12 months, with maximum mouth opening preserved and no complications recorded [30]. Indication drift is itself a safety issue, and instruments that constrain it merit the same attention as injection technique.

Patient selection also carries a conventional pharmacological dimension easily overlooked in a literature preoccupied with efficacy, and product-labelled contraindications should be considered separately from clinically relevant precautions. BoNT-A should be avoided or used only after individualized specialist assessment in patients with disorders of neuromuscular transmission, significant pre-existing dysphagia or respiratory compromise, active infection or inflammation at the injection site, or known hypersensitivity to the formulation or its excipients. Pregnancy and breastfeeding also require individualized risk-benefit assessment because evidence specific to therapeutic masticatory-muscle use is limited. Particular caution is warranted with concomitant drugs that may potentiate neuromuscular blockade, including certain aminoglycoside antibiotics and other agents affecting neuromuscular transmission.

Although immunogenic secondary non-response appears uncommon at the doses typically used for TMD, repeated exposure and increasing cumulative doses may theoretically increase the risk of neutralizing antibody development, and evidence specific to TMD populations is currently lacking [93,94,95,96] (see Table 2).

## 6. Future Perspectives and Conclusions

The position that BoNT-A currently occupies in the management of temporomandibular disorders is defined less by disagreement than by a convergence that individual conflicting trials easily obscure. The umbrella review found BoNT-A more effective than placebo for pain intensity but not superior to standard treatments, judged the risk of adverse events affecting muscle and bone to be higher than with comparator therapies, and concluded that it should be the last treatment alternative [100]. The most recent systematic review of randomized trials arrived at the same practical conclusion from a different starting point: current evidence does not support routine first-line use, but BoNT-A may be considered in selected refractory cases after conservative therapy has failed [77]. These documents are frequently cited as though they contradicted one another. All three place BoNT-A downstream of guideline-supported conservative care and reserve it for patients in whom that care has demonstrably failed.

Between what can and cannot be claimed, there is a genuine asymmetry. It cannot be said that BoNT-A outperforms standard conservative treatment [100], that superiority over saline is consistent across trials [83], that a clinically relevant dose–response relationship has been established [77], or that repeated administration over years is structurally neutral for muscle and bone [79,93]. The comparison with saline deserves a caveat of its own: intramuscular injection of saline into a tender masticatory muscle is not an inert procedure, and part of the difficulty in demonstrating superiority may partly reflect a non-inert needling effect, although the magnitude of this contribution is unknown [83]. The deeper asymmetry, however, is temporal.

The practical implication is that BoNT-A is best understood as a selected adjunct within a multimodal rehabilitation strategy rather than as a substitute for first-line conservative treatment. In clinical practice, injection should therefore be accompanied by continued therapeutic exercise, education, self-management, management of parafunctional behaviors, and behavioural or psychological interventions when indicated. Injecting a patient who has been withdrawn from these interventions would effectively convert an adjunctive treatment into monotherapy, for which current evidence is insufficient.

One consideration specific to this population deserves explicit statement. The scarcity of alternatives above first-line management, rather than the strength of the supporting evidence, probably accounts for a substantial part of the current enthusiasm for BoNT-A. Recognizing this makes the case for disciplined patient selection stronger rather than weaker, since the alternatives to injecting are not more invasive procedures but diagnostic reassessment, management of contributing comorbidities and further optimization of multidisciplinary conservative care.

Beyond the individual patient, the field would be well served by a shift in priorities away from further placebo-controlled efficacy trials, of which there are now many, towards phenotype-stratified comparative trials with long-term structural safety endpoints. At the level of the individual consultation, shared decision-making should weigh the probability and expected duration of pain relief explicitly against functional, structural, financial and treatment-burden considerations. Until such data are available, BoNT-A may remain a defensible option for a narrowly selected group of patients who have not responded to better-supported alternatives, whereas its routine use outside this context cannot currently be supported.

## Figures and Tables

**Table 1:** Grading of evidence considering most recent systematic review.

Clinical Question	Certainty	Clinical Interpretation
Reduction in masticatory muscle activity	High	BoNT-A reliably produces neuromuscular inhibition
Reduction in bite force	High	Consistently demonstrated in controlled studies
Pain reduction versus placebo	Moderate	Favorable signal but substantial heterogeneity
Superiority over conservative treatment	Low–moderate	Not consistently demonstrated
Benefit in persistent myogenous TMD	Low–moderate	Most promising clinical phenotype
Benefit in predominantly intra-articular TMD	Low	Insufficient evidence
Identification of responders	Low	No validated prediction model
Optimal dose	Low	No reproducible dose–response relationship
Duration of clinical benefit	Moderate	Generally several weeks to months
Short-term safety	Moderate	Mostly transient effects when appropriately administered
Long-term muscle effects	Moderate	Biological effects established; clinical significance incompletely defined
Mandibular skeletal effects	Low–moderate	Human studies exists, but clinical relevance remains uncertain
Use as first-line treatment	Moderate–high against	Current guideline does not support routine first-line use

These recommendations represent a research agenda proposed by the present authors rather than established consensus standards; the thresholds given for treatment cycles, follow-up duration and registry length are illustrative rather than validated.

**Table 2:** Adverse events reported after botulinum toxin type A injection into the masticatory muscles, with their relationship to dose and technique, reversibility and available mitigation strategies.

Adverse Event	Reported Frequency and Evidence	Relationship to Dose or Technique	Reversibility	Clinical Mitigation	References
Injection-site pain and local tenderness	Most frequently reported event; 20% in one sleep bruxism trial; no significant difference vs. placebo in meta-analysis	Technique-related (needle trauma, number of sites)	Days	Fine-gauge (30 G) needle; immediate local compression after injection [84]	[77,78,80]
Transient headache	Occasional across randomized trials	Not established	Days to weeks	Usually self-limiting; no specific intervention established	[77,80]
Dysphagia and regional discomfort	Uncommon; transient	Diffusion into adjacent musculature	Days to weeks	Inject at appropriate depth within muscle boundaries; avoid excessive infiltration volume [84]	[77,80]
Reduced electromyographic (EMG) activity of injected muscles	Reported in all studies that measured it	Dose-related	1–6 months	Lowest effective dose; ultrasound or electromyographic guidance improves placement accuracy, although an effect on adverse events is unproven [84]	[27,82,83]
Reduced occlusal (bite) force	Reported in all four studies that assessed it; pooled mean difference −30.3 kg at 3 months	Probably dose-related	Months	Lowest effective dose; minimize cumulative bilateral dose where clinically feasible; record bite force where feasible	[82,83]
Reduced masticatory performance and perceived chewing weakness	Transient decline after single session; more pronounced after repeated sessions	Dose- and repetition-related	Weeks to months	Lowest effective dose; avoid excessive infiltration; guidance may improve placement accuracy [84]	[27,83,90]
Masseter and temporalis atrophy (thinning)	Detectable within 2 weeks; maximal by 1 month; attenuates after 3 months	Dose- and repetition-related	Not different from baseline at 72 months after a single application in one small uncontrolled long-term sample; recovery after repeated cycles not established	Lowest effective dose; ultrasonographic assessment of muscle thickness where available, which is not an established standard; avoid short inter-injection intervals	[27,87,88,97]
Compensatory temporalis thickening	Significant increase in thickness, inversely related to masseter reduction	Follows masseter-only injection	Not characterized	Assess injected and non-injected masticatory muscles longitudinally; the clinical significance and optimal management of compensatory changes remain uncertain	[85,86,98]
Altered smile and facial asymmetry	15.4% in one sleep bruxism trial	Diffusion into mimetic muscles; technique-related	Weeks to months	Inject well within the masseter, taking the tragus–oral commissure line as the superior margin; identify borders by palpation during clenching [84]	[80,81]
Risorius paralysis	Single case report during a randomized trial	Diffusion; technique-related	Transient	As above; avoid superficial and antero-superior needle placement [81,84]	[81]
Mandibular cortical thinning	≈6% decrease in pooled human data; not confirmed in randomized computed tomography study	Possible dose relationship (temporalis dose vs. trabecular density)	Unknown	Assess baseline skeletal risk and avoid high cumulative exposure in patients at higher risk; no evidence-based imaging-surveillance protocol has been established [84,99]	[79,91,92,93]
Reduced condylar and coronoid process bone volume	Dose-related in one randomized trial; not reproduced in larger controlled studies	Dose-related; greater mandibular angle volume change after two applications than after one in pooled human data	Unknown	Lowest effective dose; record cumulative dose and interval for every cycle	[90,91,92]
Immunogenic secondary non-response	Not reported in TMD populations; frequency varies by indication and formulation (13.9% neutralizing-antibody positivity in a long-term neurological cohort, a figure not transferable to TMD dosing regimens)	Cumulative dose, single dose per cycle and short inter-injection intervals	Variable	Avoid booster injections and short intervals; lowest effective dose per cycle [95]	[94,95,96]

Entries in the References column support the reported frequency and evidence; citations within the Clinical mitigation column support the specific technique referred to. Mitigation strategies that are not supported by comparative clinical data are identified as such.

## Data Availability

No new data were created or analyzed in this study. Data sharing is not applicable to this article.
